# heredERA Breast Cancer: a phase III, randomized, open-label study evaluating the efficacy and safety of giredestrant plus the fixed-dose combination of pertuzumab and trastuzumab for subcutaneous injection in patients with previously untreated HER2-positive, estrogen receptor-positive locally advanced or metastatic breast cancer

**DOI:** 10.1186/s12885-024-12179-9

**Published:** 2024-05-24

**Authors:** Sherko Kuemmel, Catherine Harper-Wynne, Yeon Hee Park, Fábio Franke, Michelino de Laurentiis, Eva Schumacher-Wulf, Daniel Eiger, Sarah Heeson, Andrés Cardona, Özgür Özyilkan, Flavia Morales-Vàsquez, Ciara Metcalfe, Marc Hafner, Eleonora Restuccia, Joyce O’Shaughnessy

**Affiliations:** 1 Breast Unit, Kliniken Essen Mitte, Essen, Germany; 2 Department of Gynecology with Breast Center, Charité – Universitätsmedizin Berlin, Berlin, Germany; 3 Kent Oncology Centre, Maidstone Hospital, Maidstone, Kent UK; 4 Samsung Medical Center, Sungkyunkwan University, School of Medicine, Seoul, Republic of Korea; 5 Oncosite, Centro de Pesquisa Clínica Em Oncologia, Ijuí, Brazil; 6 Istituto Nazionale Tumori IRCCS “Fondazione Pascale”, Napoli, Italy; 7 Mamma Mia! Breast Cancer Magazine, Cologne, Germany; 8 F. Hoffmann-La Roche Ltd, Basel, Switzerland; 9 Roche Products Limited, Welwyn Garden City, UK; 10 Baskent University Hospital, Adana, Turkey; 11 FUCAM, A.C and Instituto Nacional de Cancerologia, Mexico City, Mexico; 12 Genentech, Inc., South San Francisco, CA USA; 13 Baylor University Medical Center, Texas Oncology, US Oncology, 3410 Worth Street, Suite 400, Dallas, TX 75246 USA

**Keywords:** Giredestrant, Pertuzumab, Trastuzumab, HER2-positive, Estrogen receptor-positive, Breast cancer

## Abstract

**Background:**

HER2-positive, estrogen receptor-positive breast cancer (HER2+, ER+ BC) is a distinct disease subtype associated with inferior response to chemotherapy plus HER2-targeted therapy compared with HER2+, ER-negative BC. Bi-directional crosstalk leads to cooperation of the HER2 and ER pathways that may drive treatment resistance; thus, simultaneous co-targeting may optimize treatment impact and survival outcomes in patients with HER2+, ER+ BC.

First-line (1L) treatment for patients with HER2+ metastatic BC (mBC) is pertuzumab, trastuzumab, and taxane chemotherapy. In clinical practice, dual HER2 blockade plus a fixed number of chemotherapy cycles are given as induction therapy to maximize tumor response, with subsequent HER2-targeted maintenance treatment given as a more tolerable regimen for long-term disease control. For patients whose tumors co-express ER, maintenance endocrine therapy (ET) can be added, but uptake varies due to lack of data from randomized clinical trials investigating the superiority of maintenance ET plus dual HER2 blockade versus dual HER2 blockade alone. Giredestrant, a novel oral selective ER antagonist and degrader, shows promising clinical activity and manageable safety across phase I–II trials of patients with ER+, HER2-negative BC, with therapeutic potential in those with HER2 co-expression.

**Methods:**

This phase III, randomized, open-label, two-arm study aims to recruit 812 patients with HER2+, ER+  locally advanced (LA)/mBC into the induction phase (fixed-dose combination of pertuzumab and trastuzumab for subcutaneous injection [PH FDC SC] plus a taxane) to enable 730 patients to be randomized 1:1 to the maintenance phase (giredestrant plus PH FDC SC or PH FDC SC [plus optional ET]), stratified by disease site (visceral versus non-visceral), type of LA/metastatic presentation (de novo versus recurrent), best overall response to induction therapy (partial/complete response versus stable disease), and intent to give ET (yes versus no). The primary endpoint is investigator-assessed progression-free survival. Secondary endpoints include overall survival, objective response rate, clinical benefit rate, duration of response, safety, and patient-reported outcomes.

**Discussion:**

heredERA BC will address whether giredestrant plus dual HER2 blockade is superior to dual HER2 blockade alone, to inform the use of this combination in clinical practice for maintenance 1L treatment of patients with HER2+, ER+ LA/mBC.

**Trial registration:**

ClinicalTrials.gov, NCT05296798; registered on March 25, 2022. Protocol version 3.0 (November 18, 2022). Sponsor: F. Hoffmann-La Roche Ltd, Grenzacherstrasse 124 4070, Basel, Switzerland.

**Supplementary Information:**

The online version contains supplementary material available at 10.1186/s12885-024-12179-9.

## Background

### Targeting the HER2 pathway

Approximately 15–20% of invasive breast cancers (BCs) are human epidermal growth factor receptor 2-positive (HER2 +) [1], an aggressive clinical subtype [2] that, prior to the availability of HER2-targeted therapies, was associated with poor prognosis and worse survival outcomes compared with HER2-negative (HER2–) BC [1–5]. The HER2-specific monoclonal antibody, trastuzumab [6, 7], transformed the treatment landscape for HER2+ BC, and together with subsequently developed HER2-targeting agents (pertuzumab, ado-trastuzumab emtansine, lapatinib, neratinib, trastuzumab deruxtecan, tucatinib, and margetuximab) are widely recommended by clinical guidelines for the treatment of patients with HER2+ BC [8–10].

Standard of care (SoC) for first-line (1L) treatment of patients with HER2+ metastatic BC (mBC) is pertuzumab, trastuzumab, and taxane chemotherapy as induction therapy, followed by maintenance treatment with pertuzumab and trastuzumab [9]. Recommendations are based on the results of the pivotal phase III CLEOPATRA trial, which demonstrated significant progression-free survival (PFS) and overall survival (OS) improvements with pertuzumab, trastuzumab, and docetaxel over trastuzumab and docetaxel [11–13]. The survival benefit with the addition of pertuzumab was maintained after more than 8 years of follow-up [14]. The phase III PERUSE trial evaluated pertuzumab and trastuzumab with investigator’s choice of taxane (paclitaxel, nab-paclitaxel, or docetaxel). Safety and efficacy results were consistent with those of CLEOPATRA, suggesting that paclitaxel is a valid alternative to docetaxel as backbone chemotherapy [15, 16], as endorsed by current clinical guidelines [9]. Use of dual HER2 blockade with induction chemotherapy followed by maintenance HER2-targeted treatment may enhance clinical benefit overall, by first maximizing tumor reduction with a fixed number of cycles of initial chemotherapy in combination with dual HER2 blockade, then switching to maintenance HER2-targeted therapy alone for better long-term tolerability [11–13, 15, 16].

More recently, a fixed-dose combination of pertuzumab and trastuzumab for subcutaneous (PH FDC SC) injection has been developed. The phase III FeDeriCa trial demonstrated that PH FDC SC provides non-inferior pertuzumab and trastuzumab serum trough concentrations (C_trough_) compared with intravenous (IV) pertuzumab and trastuzumab, with a safety profile consistent with the known safety profiles of IV pertuzumab and trastuzumab [17]. PH FDC SC offers faster and less invasive administration of pertuzumab and trastuzumab, and greater convenience, compared with the IV formulations [17]. Patient preference for PH FDC SC compared with IV pertuzumab and trastuzumab was assessed in the randomized, open-label, phase II PHranceSCa trial. Most patients in the study preferred PH FDC SC administration over the IV formulations, with the main reasons being reduced time in the clinic and greater comfort during administration [18]. The majority of healthcare professionals in PHranceSCa felt that switching from IV to SC formulations would reduce time and resource use [18]; furthermore, the phase IIIb PHaTiMa time and motion study demonstrated significant time-savings and reduction in resources and consumables used with PH FDC SC compared with IV pertuzumab and trastuzumab or IV pertuzumab and SC trastuzumab [19]. PH FDC SC is approved by the US Food and Drug Administration (FDA; approval allows for at-home administration following completion of chemotherapy [20]), the European Medicines Agency (EMA), and other regulatory authorities for the same indications as IV pertuzumab and trastuzumab [21, 22].

### Targeting the hormone receptor pathway

The therapeutic mainstay for hormone receptor-positive BCs (which account for ~70–80% of diagnosed BCs [23, 24]) is endocrine therapy (ET) with or without targeted therapies; in the metastatic setting, chemotherapy is reserved for later lines of treatment or for patients with visceral crisis [8, 9]. Current approved ETs include aromatase inhibitors (AIs) such as anastrozole, letrozole, and exemestane, the selective estrogen receptor (ER) modulator tamoxifen, the selective ER antagonist and degrader (SERD) fulvestrant and, more recently, the oral SERD elacestrant [25–27]. However, despite the effectiveness of ETs, most patients with mBC will eventually develop progressive disease due to primary or secondary ET resistance [28]. Fulvestrant has shown superior efficacy versus anastrozole in patients with 1L ER+, HER2– locally advanced (LA)/mBC [29]. While fulvestrant has some activity in *ESR1*-mutated tumors [30] (a common mechanism of acquired resistance to ET [31]), it also has unfavorable bioavailability and pharmacokinetics, requiring repeated intramuscular injections [32, 33]. Therefore, oral SERDs with greater convenience of administration and more potent activity are needed in this setting. Elacestrant, a next-generation oral SERD, was recently granted FDA-approval; however, at the time of writing this manuscript, it is indicated only in the endocrine-resistant setting for patients with *ESR1* mutations [27].

### Giredestrant: a novel, next-generation ET

Giredestrant is a highly potent, non-steroidal, next-generation, oral SERD that achieves robust ER occupancy and is effective regardless of *ESR1* mutation status [34, 35]. Preclinical data show that giredestrant has higher in vitro potency compared with fulvestrant, tamoxifen, and other oral SERDs in ER+ BC cell lines [33, 35]. Phase I–II clinical studies have demonstrated that giredestrant has promising clinical and pharmacodynamic activity, as monotherapy and in combination with the cyclin-dependent kinase 4/6 (CDK4/6) inhibitor palbociclib, and is well tolerated by patients with ER+, HER2– BC [34, 36–43]. Giredestrant is currently being evaluated in several phase III trials as a potential endocrine backbone therapy of choice [44–46].

### Addressing the unmet need in HER2+, ER+ BC

**Fig. 1:**
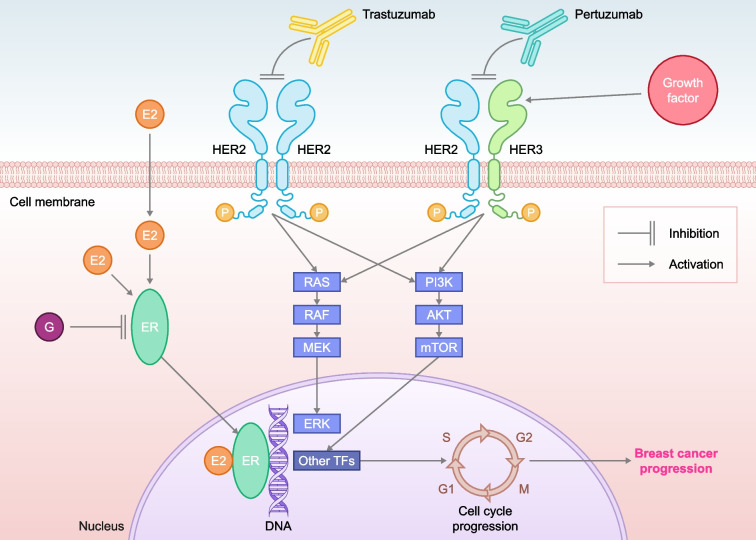
Bi-directional crosstalk between the HER2 and ER pathways. E2: estradiol, ER: estrogen receptor, G: giredestrant, HER: human epidermal growth factor receptor, TF: transcription factor

Approximately 50–60% of patients with HER2+ BC have tumors that are also ER+; HER2+, ER+ BC is considered a distinct disease subtype from HER2+, ER-negative BC [47–50]. At a molecular level, according to the PAM50 classification, HER2+, ER– BC is more frequently HER2-enriched, whilst HER2+, ER+ BC is more frequently luminal, underscoring intrinsically different disease biology according to ER status [49]. Patients with HER2+, ER+ BC tend towards later disease recurrence [51], have a higher frequency of bone metastasis [47], and have a poorer response to chemotherapy plus HER2-targeted therapies, when compared with HER2+, ER– BC [11, 17, 52, 53], suggesting the ER pathway acts as an escape mechanism promoting tumor survival under sustained HER2 inhibition [47, 54]. Conversely, resistance to ETs in HER2+, ER+ BC may arise through hyperactive HER2-mediated signaling [54]. HER2 and ER are the main drivers of cell proliferation and survival in BC [54], and bi-directional crosstalk can lead to cooperation between these two pathways in HER2+, ER+ BC (Fig. 1) and development of treatment resistance; moreover, inhibition of one pathway leads to upregulation of the other [48, 54, 55]. Thus, simultaneous co-targeting may optimize treatment impact and survival outcomes in patients with HER2+, ER+ BC [48, 54, 55].

Following the results of the pivotal CLEOPATRA trial, dual HER2 blockade with pertuzumab and trastuzumab combined with taxane induction therapy is SoC for the 1L treatment of patients with HER2+ mBC [9]. In clinical practice, after achieving clinical benefit with an average of six cycles of pertuzumab and trastuzumab plus taxane induction, maintenance therapy is continued with pertuzumab and trastuzumab only, until disease progression or unmanageable toxicity [50]. However, the CLEOPATRA trial design precluded the use of ET in conjunction with maintenance pertuzumab and trastuzumab in patients whose tumors were also ER+ [11]. A pivotal trial of second-line (2L) treatment with an antibody–drug conjugate in HER2+ mBC also did not assess the use of ET in combination with the HER2-targeted therapy [56]. Consequently, data are lacking from prospective, randomized clinical trials assessing the superiority of the addition of ET to dual HER2 blockade with trastuzumab and pertuzumab versus dual HER2 blockade alone in the maintenance setting. Therefore, although maintenance ET plus pertuzumab and trastuzumab is endorsed by clinical guidelines, the uptake of ET has been shown to vary [57, 58].

Preclinical data provide proof-of-concept for combining HER2-targeted therapy with ET in HER2+, ER+ BC [59]. There is also clinical evidence from phase II–III trials demonstrating the synergy and tolerability between ET and single or dual HER2 blockade [60]; data include, but are not limited to, the TAnDEM, EGF 30008, and eLEcTRA trials, which demonstrated a PFS/time to progression treatment benefit with ET plus single-agent HER2-targeted therapy versus ET alone (hazard ratios [HR] of 0.63–0.71) [61–63], and the ALTERNATIVE and PERTAIN trials, which showed significantly superior PFS with ET plus dual HER2 blockade versus ET plus single-agent HER2-targeted therapy (HR 0.62–0.65) [64, 65]. Taken together, these results support the rationale for co-targeting the HER2 and ER pathways in the heredERA BC study.

### heredERA BC study

**Fig. 2:**
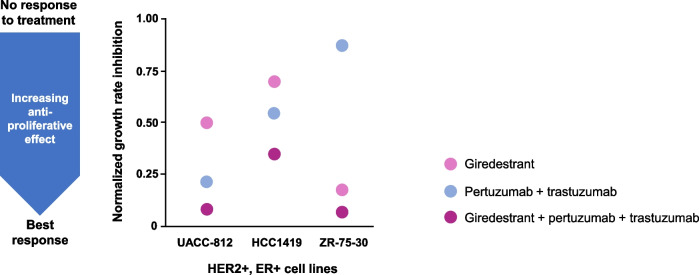
Combined treatment with giredestrant and HER2-targeted therapy (pertuzumab and trastuzumab) shows greater antiproliferative effect than either treatment alone (F. Hoffmann-La Roche Ltd. Personal communication, unpublished data). Cell growth rate inhibition of ‘1’ denotes no effect on growth whereas ‘0’ represents stasis of the cell population. ER+: estrogen receptor-positive, HER2+: human epidermal growth factor receptor 2-positive

The PERTAIN trial demonstrated that dual HER2 blockade with pertuzumab and trastuzumab plus ET is effective in HER2+, ER+ mBC; although, the study did not evaluate whether the addition of ET to HER2-targeted dual blockade was more effective than dual blockade alone following chemotherapy induction [65]. Based on those results, ET may be given with pertuzumab and trastuzumab at chemotherapy discontinuation, as endorsed by clinical guidelines [9]. Non-randomized, retrospective data [50, 66] also appear to support the use of maintenance ET plus dual HER2 blockade. Moreover, exploratory clinical data have shown that HER2+ tumors exposed to HER2-targeted treatment ± chemotherapy frequently experience an intrinsic molecular subtype shift from HER2-enriched to luminal subtypes, thus highlighting the relevance of adding maintenance ET to increase treatment efficacy [67, 68]. Preclinical data (Fig. 2) have shown that the combination of giredestrant, pertuzumab, and trastuzumab resulted in a greater anti-proliferative treatment effect (as measured by normalized growth rate inhibition [69]) in HER2+ ER+ cell lines than either giredestrant or pertuzumab and trastuzumab alone (F. Hoffmann-La Roche Ltd. Personal communication, unpublished data). Importantly, there are no expected major overlapping toxicities between giredestrant and PH FDC SC based on current knowledge of the safety profiles of each individual drug. Trials of trastuzumab ± pertuzumab in combination with ET have shown that the simultaneous targeting of the HER2 and ER pathways is feasible, with no major safety signals reported for the treatment combination arms [61, 65].

heredERA BC is a phase III, randomized, open-label, two-arm study. The primary objective will be to assess whether addition of giredestrant to SoC HER2-targeted therapy (PH FDC SC) has superior efficacy outcomes compared with PH FDC SC, following an induction therapy phase with PH FDC SC plus a taxane. Secondary objectives include additional efficacy assessments and safety. Exploratory objectives will assess patient-reported outcomes (PROs), pharmacokinetics, and biomarkers. PH FDC SC was selected as the comparator treatment given its non-inferior C_trough_ to the IV formulation, considerable shorter administration time [17], and possibility for at-home administration [70] with the potential to improve the patient experience, especially when used in combination with an oral ET (giredestrant).

## Methods

### Study setting

**Fig. 3:**
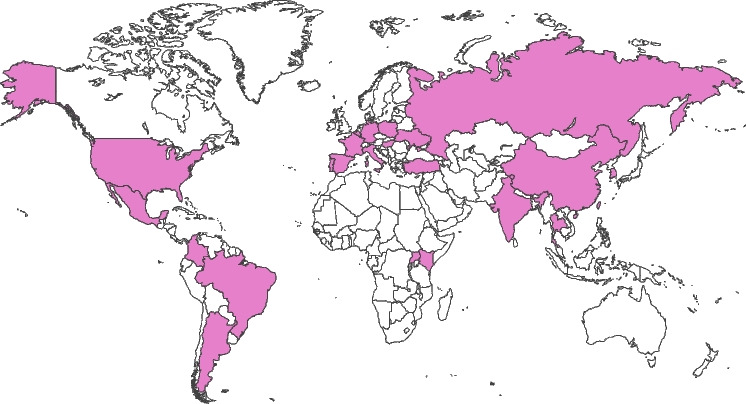
Participating countries. The heredERA BC study is being conducted across 224 sites in 24 countries. BC: breast cancer

heredERA BC is a global study that is being conducted across 224 sites in 24 countries (Fig. 3). A full list of participating study sites and countries can be found at ClinicalTrials.gov (NCT05296798). The study is currently recruiting, and the first patient was enrolled on July 18, 2022.

### Eligibility criteria

Eligible patients must have confirmed HER2+, ER+ LA or mBC not amenable to curative resection, with at least one measurable lesion and/or non-measurable disease evaluable according to Response Evaluation Criteria in Solid Tumors (RECIST) version 1.1 [71]. Key inclusion and exclusion criteria are shown in Table 1.

**Table 1:** Key inclusion and exclusion criteria

**Key inclusion criteria**	**Key exclusion criteria**
• Histologically or cytologically confirmed and documented ER+, HER2+ adenocarcinoma of the breast with metastatic or locally advanced disease not amenable to curative resection • Disease-free interval of ≥ 6 months • ECOG PS 0–1 • LVEF of ≥ 50% measured by echocardiogram or multiple-gated acquisition scan • Adequate hematologic and end-organ function	• Previous systemic non-hormonal anticancer therapy for advanced or metastatic breast cancer (up to one line of single-agent ET in the locally advanced/metastatic setting is allowed) • Previous treatment with a SERD • Active uncontrolled or symptomatic CNS metastases, carcinomatous meningitis, or leptomeningeal disease • Investigational therapy in the 28 days prior to initiation of induction therapy • Localized palliative radiotherapy in the 14 days prior to initiation of induction therapy • Active cardiac disease, history of cardiac dysfunction, or poorly controlled hypertension • History of other malignancy in the 5 years prior to screening with the exception of the cancer under investigation in this study and malignancies with a negligible risk of metastasis or death • Men and pre-/perimenopausal women: known hypersensitivity to LHRH agonist or unwilling to undergo and maintain LHRH agonist treatment for the duration of ET that requires gonadal function suppression
**Maintenance phase inclusion criteria**	
• Achieve a minimum of SD according to RECIST v1.1. (after ≥ 4 induction cycles) • LVEF of ≥ 50%	

*Abbreviations*: *CNS* Central nervous system, *ECOG PS* Eastern Cooperative Oncology Group performance score, *ER+* Estrogen receptor-positive, *ET* Endocrine therapy, *HER2+* Human epidermal growth factor receptor 2-positive, *LHRH* Luteinizing hormone releasing hormone, *LVEF* Left ventricular ejection fraction, *RECIST* Response Evaluation Criteria in Solid Tumors, *SD* Stable disease, *SERD* Selective estrogen receptor antagonist and degrader

### Randomization and treatment allocation

**Fig. 4:**
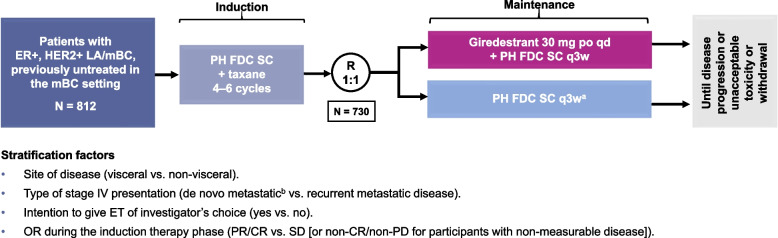
Study design. CR: complete response, ER+: estrogen receptor-positive, ET: endocrine therapy, HER2+: human epidermal growth factor receptor 2-positive, LA: locally advanced, mBC: metastatic breast cancer, OR: overall response, PD: disease progression, PH FDC SC: fixed-dose combination of pertuzumab and trastuzumab for subcutaneous injection, po: orally, PR: partial response, qd: once daily, q3w: once every 3 weeks, R: randomization, SD: stable disease. ^a^ Endocrine therapy (aromatase inhibitor/tamoxifen) will be allowed in the PH FDC SC arm. ^b^ Breast cancer that presents as stage IV disease at first diagnosis

The full study schema is shown in Fig. 4. Patients will first be enrolled into the induction phase to receive four to six cycles of PH FDC SC and a taxane; those who tolerate six cycles of induction therapy and do not experience disease progression (PD) may, at the investigator’s discretion, receive up to two additional cycles (maximum total of eight, per SoC). PH FDC SC (containing pertuzumab 1,200 mg, trastuzumab 600 mg, and rHuPH20 30,000 units in the loading dose, and pertuzumab 600 mg, trastuzumab 600 mg, and rHuPH20 20,000 units in subsequent doses) will be administered once every 3 weeks and prior to taxane chemotherapy (investigator’s choice of docetaxel or paclitaxel, administered according to local prescribing information). Patients who have received a taxane prior to enrollment will receive the same taxane during the induction phase. In the maintenance phase, patients will be randomized 1:1 to receive giredestrant (30 mg taken orally once daily, on Days 1–21 of each 21-day cycle) and PH FDC SC or PH FDC SC. Randomization will be stratified according to site of disease (visceral versus non-visceral), type of locally advanced/metastatic presentation (de novo versus recurrent), best overall response to induction therapy (partial or complete response [PR/CR] versus stable disease [or non-CR/non-PR for those with non-measurable disease]), and intent to give ET of investigator’s choice (yes versus no). Patients in the PH FDC SC arm may receive optional ET (tamoxifen or an AI [anastrozole, letrozole, or exemestane]) administered according to local prescribing information. The decision to use optional ET will be made prior to randomization. Pre-/perimenopausal women and men, who are receiving treatment with giredestrant or ET will receive a luteinizing hormone-releasing hormone (LHRH) agonist. Study treatment will be administered until PD (per RECIST v1.1), limiting toxicity, death, or withdrawal of consent.

Dose modifications for PH FDC SC and giredestrant are not permitted; taxane dose modifications are per SoC. Concomitant therapies are permitted, with the exception of other investigational therapies, anticancer therapies, regular systemic steroidal treatment (except short-term corticosteroids for allergic or infusion reactions), tumor necrosis factor-α inhibitors, anti-T cell antibodies, systemically active oral, injected, or implanted hormonal contraception (except for previously implanted, progesterone-coated, intrauterine devices), hormone-replacement therapy, and strong CYP3A inhibitors or inducers.

Patients who are being dosed at a study site will receive the study drug under supervision, and dosing details will be recorded in the electronic Case Report Form (eCRF). Compliance with study treatment will be assessed for patients who self-administer study treatment at home. Patients will be required to complete a medication diary; for any study drug administered at home, the medication diary, unused study drug, and study drug containers (used or unused) will be collected and reviewed for drug accountability at the start of each cycle.

### Outcome measures and endpoints

The primary and secondary efficacy endpoints are listed in Table 2. Safety endpoints include incidence and severity of adverse events, with severity determined according to the National Cancer Institute’s Common Terminology Criteria for Adverse Events Version 5.0 (NCI-CTCAE v5.0), and the change from baseline to targeted clinical laboratory test results. Exploratory endpoints include health-related quality of life, PROs, biomarkers, and pharmacokinetics.

**Table 2:** Study efficacy endpoints

**Endpoint**	**Timing**
**Primary**	
PFS, defined as the time from randomization to the first occurrence of disease progression or death from any cause^a^	Patients will undergo tumor assessments at screening, at Week 9^b^ (to ensure at least one evaluation is conducted during the induction therapy phase), then every 12 weeks up to 36 months, then every 18 weeks thereafter, regardless of dose delays, until radiographic disease progression per RECIST v1.1
**Secondary**	
OS, defined as the time from randomization to death from any cause	
ORR (following randomization), defined as the proportion of patients with a CR or PR on two consecutive occasions ≥ 4 weeks apart^a^	
DoR (following randomization), defined as the time from the first occurrence of a documented objective response to disease progression or death from any cause^a^	
CBR (following randomization), defined as the proportion of patients with SD for ≥ 24 weeks or a CR or PR^a^	
Mean and mean changes from baseline score in function (role, physical) and HRQoL by cycle and between treatment arms as assessed through the use of the functional and GHS/QoL scales of the EORTC QLQ-C30	Patients will compete questionnaires at baseline (Cycle 1, Day 1 of maintenance therapy) and at subsequent timepoints

*Abbreviations*: *CBR* Clinical benefit rate, *CR* Complete response, *DoR* Duration of response, *EORTC QLQ-C30* European Organisation for the Research and Treatment of Cancer Quality of Life–Core 30 Questionnaire, *GHS* Global health status, *HRQoL* Health-related quality of life, *ORR* Objective response rate, *OS* Overall survival, *PFS* Progression-free survival, *PR* Partial response, *QoL* Quality of life, *RECIST* Response Evaluation Criteria in Solid Tumors, *SD* Stable disease

^a^Investigator-assessed, according to RECIST v1.1

^b^Weeks counted from administration of first induction therapy, on or off study, whichever is earlier

### Study assessments

The schedule of study activities is shown in Table 3.

**Table 3:** Schedule of activities

	***Screening***^***a***^		***Induction therapy cycles***^***b***^*** (21-day cycles)***	***Maintenance therapy cycles***^***c***^*** (21-day cycles)***	***Treatment discontinuation***	***Long-term follow-up (q3m)***^***d***^
	***–28 days to enrollment***	***–7 days to enrollment***	***Day 1 (± 3 days)***	***Day 1 (± 3 days)***	***28 days after final dose of PH FDC SC (± 3 days)***	***(± 15 days)***
**Informed consent**	X					
***Tumor tissue submission for prospective HER2 status assessment (central laboratory)***	X (may be performed outside of the 28-day screening window)					
***Tumor assessment***^e^	X		X 9 weeks after first induction therapy (on or off study), then q12w for 36 months, q18w thereafter. A window of ± 7 days is allowed		X^e^	
***ECHO or MUGA scan***^f^	X		X Every four cycles, including any off-study induction cycles		X	X
***ECOG PS***	X		X	X^g^	X	
**Interventions:**						
***PH FDC SC administration***			X	X^g,h^		
***Taxane administration***			X			
***Giredestrant administration***				X (qd Days 1–21 of each cycle)		
***Optional ET administration***				X (administered to applicable patients in the PH FDC SC arm only)		
***LHRH agonist administration (pre-/perimenopausal women, and men, in the giredestrant arm and in the PH FDC SC arm if receiving ET)***				X (administered to applicable patients q4w^g^)		
**Assessments:**						
***Adverse events***^i^	X	X	X	X	X	X
***Survival and anticancer therapy follow-up***					X	X

*ECG* Electrocardiogram, *ECHO* Echocardiogram, *ECOG PS* Eastern Cooperative Oncology Group performance status, *ET* Endocrine therapy, *HER2* Human epidermal growth factor receptor 2, *IV* Intravenous, *LHRH* Luteinizing hormone-releasing hormone, *LVEF* Left ventricular ejection fraction, *MUGA* Multiple-gated acquisition scan, *PH FDC SC* Fixed-dose combination of pertuzumab and trastuzumab for subcutaneous injection, *PK* Pharmacokinetic, *qd* Once daily, *q3m* Once every 3 months, *q4w* Once every 4 weeks, *q12w* Once every 12 weeks, *q18w* Once every 18 weeks, *SC* Subcutaneous

^a^Results of standard-of-care assessments performed prior to obtaining informed consent and which fall into the specified screening window may be used; such tests do not need to be repeated for screening

^b^During the induction-therapy phase, all patients will receive PH FDC SC in combination with a taxane (i.e., docetaxel or paclitaxel) for four to six cycles, as per the standard of care. At the investigator’s discretion, patients who tolerate six cycles of induction therapy may be given up to two additional cycles of the same taxane + PH FDC SC, for a total of up to eight cycles. Patients who have received one or two cycles of PH FDC SC (or trastuzumab SC with IV pertuzumab, or IV pertuzumab and trastuzumab) with docetaxel or paclitaxel prior to enrollment are eligible and these additional cycles will count towards eligibility for the maintenance phase

^c^Following the induction therapy phase, eligible patients will be randomized to one of two treatment arms. A 7-day window is permitted from randomization to Day 1 of the first cycle of study treatment in the maintenance phase

^d^Follow-up visits are based on the date of the last dose of PH FDC SC and not on treatment discontinuation visit, (i.e., 3-month follow-up visit is 3 months after the date of final dose of PH FDC SC) with 1 month equal to 30 days. Follow-up visits are to be performed within ± 15 days. Patients who discontinue therapy in the induction-therapy phase will enter into follow-up and only be followed for LVEF assessments, pregnancy testing, and long-term adverse events

^e^Tumor assessments should be scheduled relative to the date of administration of the first induction therapy (on or off study, whichever is earlier), not the date of the previous tumor assessment. Patients who discontinue study treatment for any reason other than disease progression or death should continue to undergo tumor assessments as per the schedule, even if they start new anticancer therapy. Tumor assessment at the treatment discontinuation visit is not required for these patients

^f^ECHO or MUGA should be obtained during the last week (Days 15–21) of the third induction therapy cycle (i.e., during the week prior to the fourth cycle [including any off-study induction cycles] to allow evaluation of the results before the next treatment cycle), and every fourth cycle thereafter. More frequent ECHO or MUGA assessments can be performed as clinically indicated. If not performed within the previous 6 weeks, ECHO or MUGA should be obtained at the treatment discontinuation visit. During the follow-up period, ECHO or MUGA should be obtained at 6 months and 12 months, and then annually thereafter (± 28 days). Patients who discontinue study treatment for heart failure or LVEF decline should continue to undergo LVEF assessments according to this schedule, irrespective of the initiation of alternative systemic anticancer therapy, until event resolution, improvement to baseline status, no further improvement can be expected, or death

^g^For patients at participating sites who have provided written informed consent to participate in mobile nursing visits, this assessment or procedure may be performed by a trained nursing professional at the patient's home or another suitable location (if allowed by country regulations), only for those visits where the participant is not required to attend the clinic for biomarker or PK sampling or ECG. On visits that require biomarker or PK sampling, or ECG to be performed, all assessments should be performed at the clinic

^h^PH FDC SC may be administered at the patient’s home or another suitable location in the countries where this is possible per country regulations. If PH FDC SC is administered outside of the clinic, then the Day 1 giredestrant dose for that cycle may also be administered outside of the clinic

^i^After informed consent has been obtained but prior to enrollment and initiation of induction therapy, only serious adverse events caused by a protocol-mandated intervention should be reported. After initiation of induction therapy, all adverse events will be reported until the treatment discontinuation visit (28 days after the final dose of study treatment). After this period, all deaths, regardless of cause, should be reported. In addition, the Sponsor should be notified if the investigator becomes aware of any serious adverse event that is believed to be related to prior exposure to study drug. Heart failure (irrespective of causal relationship and for up to 3 years after PH FDC SC discontinuation), should also continue to be reported

### Sample size and statistical analysis

Approximately 812 patients will be enrolled into the induction phase, to allow for ~ 730 patients to be randomized in the maintenance phase (based on the assumption that up to ~ 10% of patients may experience PD, limiting toxicity, or withdraw consent). Patients who are still receiving induction therapy after the target N is achieved may enter the maintenance phase, if deemed eligible by the investigator. The primary endpoint of the study is investigator-assessed PFS. OS will be tested hierarchically if there is a statistical significance of PFS. Both PFS and OS will be compared between treatment arms using the stratified log-rank test and the HR estimated using a stratified Cox proportional-hazards model. For each treatment arm, Kaplan–Meier methodology will be used to estimate the median, and the Brookmeyer–Crowley method will be used to construct the 95% confidence intervals.

### Data collection, management, and analysis

All patient data relating to the study will be recorded in eCRFs unless transmitted to the sponsor or designee electronically. The sponsor or designee is responsible for the data management of this study, including checking the quality of data. PROs will be collected using European Organisation for the Research and Treatment of Cancer (EORTC) Quality of Life–Core 30 Questionnaire, EORTC Quality of Life–BR23 Questionnaire, the worst pain item from the Brief Pain Inventory-Short Form, select items of the PRO-CTCAE, the GP5 overall treatment side-effect bother item of the Functional Assessment of Cancer Therapy–General, the Work Productivity and Activity Impairment Questionnaire: General Health, and the EuroQol 5-Dimension, 5-Level Questionnaire. Questionnaires will be completed by patients at baseline, and at predefined timepoints throughout the study.

### Data monitoring

An independent Data-Monitoring Committee will evaluate unblinded safety data on a regular basis during the study. Study monitors will perform ongoing monitoring activities and ensure the study is being conducted in accordance with the protocol and study agreements, the International Council for Harmonisation (ICH) Guideline for Good Clinical Practice, and all applicable regulatory requirements.

### Data protection

Information technology systems used to collect, process, and store study-related data are secured by technical and organizational security measures designed to protect such data against accidental or unlawful loss, alteration, or unauthorized disclosure or access. In the event of a data security breach, appropriate mitigation measures will be implemented. Patients will be assigned a unique identifier by the sponsor. Any patient records or datasets transferred to the sponsor will contain the identifier only; the patient’s name or any information that would make the patient identifiable will not be transferred. Patients will be informed that their personal study-related data will be used by the sponsor in accordance with local data protection law.

## Discussion

ER expression in HER2+ BC implies a distinct biology compared with that of HER2+, ER– BC: patients diagnosed with HER2+, ER+ BC have tumors that are less proliferative, have lower *HER2* gene amplification, and thereby are more frequently of the luminal subtype [49, 72]. Overall, clinical responses to chemotherapy with HER2-targeted therapies are also lower: in a pooled individual patient-level data analysis of five trials involving 1,763 patients, neoadjuvant trastuzumab ± pertuzumab and chemotherapy entailed lower responses rates in patients with HER2+, HR+ eBC (pathologic complete response [pCR] rate: 34.4%) compared with those with HER2+, HR– eBC (pCR rate: 55.4%) [73]. Besides, following HER2-targeted therapy ± chemotherapy, there is an increase in the prevalence of molecularly defined luminal tumors in lieu of HER2-enriched ones (“luminal shift”), underscoring increasing ER dependency [67, 68]. In this sense, co-targeting HER2 and ER may therefore help to optimize survival outcomes for patients with HER2+, ER+ BC; the heredERA BC study is aiming to address this unmet need by evaluating the combination of PH FDC SC with giredestrant as maintenance therapy, following an induction phase of PH FDC SC and a taxane.

Pertuzumab plus trastuzumab is established as SoC in 1L HER2+ mBC, and PH FDC SC provides shorter administration times, greater comfort during administration, and is preferred by patients over the IV formulations, as demonstrated in the PHranceSCa study [18]. Giredestrant has already demonstrated promising clinical and pharmacodynamic activity, and was shown to be well tolerated by patients with ER+, HER2– BC [34, 36–43]. coopERA BC was the first randomized study demonstrating superior suppression of tumor cell proliferation with an oral SERD over an AI in ER+, HER2– eBC. A greater relative geometric mean reduction of Ki67 was seen with giredestrant versus anastrozole after 2 weeks of treatment, and this effect was maintained at surgery after ET was combined with palbociclib for 16 weeks [39, 42]. The acelERA BC study in the 2L and third-line setting did not meet its primary endpoint of improved investigator-assessed PFS with giredestrant versus physician’s choice of ET (single-agent fulvestrant or an AI) [43]. However, a numerical improvement in investigator-assessed PFS was seen, with a more pronounced effect in patients with *ESR1*-mutated tumors [43], providing further proof of concept of the activity of giredestrant. The MORPHEUS BC phase I/II study is evaluating giredestrant treatment combinations; Cohort 1 includes patients with ER+, HER2– mBC. Preliminary data for giredestrant plus abemaciclib or ribociclib showed that the combinations were well tolerated, supporting the combinability of giredestrant with these CDK4/6 inhibitors [74].

Giredestrant is undergoing a broad clinical development program; in addition to the heredERA BC study, it is also being investigated as a potential endocrine backbone therapy of choice in several phase III trials of patients with ER+, HER2– BC in the early and metastatic settings. In the eBC setting, the lidERA BC study is evaluating adjuvant giredestrant versus endocrine monotherapy; in the mBC setting, persevERA BC is a study of 1L giredestrant plus palbociclib versus letrozole plus palbociclib; and evERA BC will assess giredestrant versus physician’s choice of ET, both in combination with everolimus, in patients previously treated with a CDK4/6 inhibitor plus ET [44–46]. Cohort 2 of the MORPHEUS BC study is evaluating giredestrant treatment combinations of PH FDC SC with or without a CDK4/6 inhibitors in patients with HER2+, ER+ mBC [75].

The therapeutic landscapes in HER2+ BC and ER+ BC are evolving rapidly, with a wealth of potential new treatment options that may provide optimal blockade of the HER2 and ER oncogenic drivers within HER2+, ER+ disease. Current clinical development in HER2+ BC includes studies of antibody–drug conjugates [76], tyrosine kinase inhibitors [77], PI3K inhibitors [78], and vascular endothelial growth factor inhibitors [79], whilst next-generation oral SERDs are being investigated in ER+ BC [80–91].

In clinical practice, the use of maintenance ET concomitantly with pertuzumab and trastuzumab in 1L HER2+, ER+ mBC is varied [57, 58]; potentially because no phase III trial was conducted to demonstrate that the addition of ET to dual HER2 blockade is better than dual HER2 blockade alone. In the pivotal CLEOPATRA trial, concomitant ET for patients whose tumors co-expressed ER was not permitted together with pertuzumab and trastuzumab [11]. The DESTINY-Breast-03 trial recently established T-DXd as a new SoC in 2L HER2+ mBC [9]; however, this trial also did not assess the use of ET in combination with HER2-targeted therapy [92]. Additionally, in the phase II PERTAIN trial, what was demonstrated instead is that dual HER2 blockade with pertuzumab and trastuzumab plus an AI shows greater efficacy versus single HER2 blockade with trastuzumab plus an AI [65]. In this sense, heredERA BC is one of the few trials testing a therapeutical strategy tailored by tumor biology beyond HER2 positivity. Likewise, the phase III PATINA study is evaluating the benefit of adding maintenance palbociclib to HER2-targeted therapy (trastuzumab ± pertuzumab) plus ET (AI or fulvestrant) in the 1L HER2+, ER+ mBC setting [93], while the phase II monarcHER study has demonstrated a significant PFS benefit with abemaciclib, trastuzumab, and ET versus trastuzumab plus chemotherapy (HR 0.67) in patients with HR+, HER2+ advanced BC who had received ≥ 2 HER2-targeted therapies for advanced disease [94].

The heredERA BC study will therefore provide valuable clinical evidence to inform the use of maintenance ET in the 1L HER2+, ER+ mBC setting, an area where there is unmet need but in which data are currently scarce. The study will evaluate giving a fixed number of chemotherapy cycles (induction therapy) before moving to a personalized maintenance treatment regimen, an approach that may be more efficacious due to being informed by the tumor biology of individual patients, and more tolerable and suitable for longer treatment duration due to more durable tumor control. Finally, the combination of PH FDC SC with the oral SERD giredestrant may potentially facilitate treatment outside of the hospital for patients with 1L HER2+, ER+ mBC, given that PH FDC SC is suitable for flexible care initiatives [20, 70].

In conclusion, new therapeutic strategies are needed that can co-target the HER2 and ER pathways to prevent bi-directional pathway crosstalk and the development of treatment resistance. The heredERA BC study is aiming to address this challenge through optimizing the targeting of disease biology, by evaluating the efficacy and safety of SoC HER2-targeted therapy (PH FDC SC) combined with the next-generation oral SERD giredestrant, in patients with HER2+, ER+ mBC treated in the 1L setting.

### Supplementary Information

**Supplementary Material 1.**

## Data Availability

The results of this study may be published or presented at scientific meetings. The Sponsor will comply with the requirements for publication of study results. For eligible studies qualified researchers may request access to individual patient level clinical data through a data request platform. At the time of writing this request platform is Vivli https://vivli.org/ourmember/roche/. For up-to-date details on Roche’s Global Policy on the Sharing of Clinical Information and how to request access to related clinical study documents, see here: https://go.roche.com/data_sharing. Anonymized records for individual patients across more than one data source external to Roche cannot, and should not, be linked due to a potential increase in risk of patient re-identification.

## Abbreviations

1L: First line
2L: Second line
AI: Aromatase inhibitor
BC: Breast cancer
CBR: Clinical benefit rate
CDK4/6: Cyclin-dependent kinase 4/6
CNS: Central nervous system
CR: Complete response
C_trough_: Trough concentration
DoR: Duration of response
E2: Estradiol
EC: Ethics committee
ECG: Electrocardiogram
ECHO: Echocardiogram
eCRF: Electronic case report form
ECOG PS: Eastern Cooperative Oncology Group performance score
EMA: European Medicines Agency
EORTC QLQ-C30: European Organisation for the Research and Treatment of Cancer Quality of Life–Core 30 Questionnaire
ER+: Estrogen receptor-positive
ET: Endocrine therapy
FDA: US Food and Drug Administration
G: Giredestrant
GHS: Global health status
HER2+/–: Human epidermal growth factor receptor 2-positive/-negative
HR: Hazard ratio
HRQoL: Health-related quality of life
ICH: International Council for Harmonisation
IRB: Institutional review board
IV: Intravenous
LA: Locally advanced
LHRH: Luteinizing hormone-releasing hormone
LVEF: Left ventricular ejection fraction
mBC: Metastatic breast cancer
MUGA: Multiple-gated acquisition scan
NCI-CTCAE: National Cancer Institute’s Common Terminology Criteria for Adverse Events
ORR: Objective response rate
OS: Overall survival
PD: Disease progression
PFS: Progression-free survival
PH FDC SC: Pertuzumab and trastuzumab for subcutaneous injection
PK: Pharmacokinetic
po: Orally
PR: Partial response
PRO: Patient-reported outcome
qd: Once daily
q3w: Every 3 weeks
q4w: Every 4 weeks
q12w: Every 12 weeks
q18w: Every 18 weeks
R: Randomized
RECIST: Response Evaluation Criteria in Solid Tumors
SD: Standard deviation
SERD: Selective estrogen antagonist and degrader
TF: Transcription factor
