# Supplementary material for: heredERA Breast Cancer: a phase III, randomized, open-label study evaluating the efficacy and safety of giredestrant plus the fixed-dose combination of pertuzumab and trastuzumab for subcutaneous injection in patients with previously untreated HER2-positive, estrogen receptor-positive locally advanced or metastatic breast cancer
Source: BMC Cancer. 2024 May 24;24:641. doi: 10.1186/s12885-024-12179-9 (PMC11127459; doi:10.1186/s12885-024-12179-9)
Supplement: Supplementary file 1 — Supplementary Material 1. [file 12885_2024_12179_MOESM1_ESM.docx]

# Supplementary Information

# List of ethics committees and institutional review boards

CEIC - Comissão de Ética para Investigação Clínica, CEIC - Comissão de Ética para Investigação Clínica, Av. do Brasil, 53 - Pav 17-A , Parque da Saúde de Lisboa, 1749-004, Lisboa, Portugal.

Shandong Cancer Hospital; EC, 440 Jiyan Road, 250117, Jinan, China.

CEI del Hospital Hispano; Comité de Ética en Investigación, Pedro Moreno No. 934, Col. Zona Centro, Colonia Centro, 44100, Guadalajara, Jalisco, Mexico.

Zhejiang People's Hospital; EC/IRB, No.158 Shangtang Road, Hangzhou City, Zhejiang Province, Hangzhou City, China, 310000, Hangzhou, China.

EC of Jiangsu Cancer Hospital.

EC of Hunan Cancer Hospital, No.283, Tongzipo Road, Hunan Province, 4th Floor, 15th Building, 410013, Changsha City, China.

The 900th Hospital of PLA joint service support force, No. 156, North of Xierhuan Road, Fuzhou, Fujian, 350009, Fuzhou City, China.

Comité de Ética en Investigación Hospital La Misión, Avenida del Hospital No. 112 1er y 2do piso, 64718, Monterrey Nuevo León, Nuevo Leon, Mexico.

CEI Instituto de Investigaciones Clinicas para la Salud; Comite de Etica, Patoni No. 404 Colonia Centro, 34000, Victoria De Durango, Durango, Mexico.

CEI del Instituto Tecnologico y de Estudios Superiores de Monterrey; ITESM, Av. Morones PrietoNo. 3000 Pte. Col. Los Doctores, 64710, Monterrey, Nuevo Leon, Mexico.

CEI del Centro Estatal de Cancerologia; Comite de Etica en Investigacion, Ejército Mexicano #3700, Colonia Centro, 31000, Chihuahua, Chihuahua, Mexico.

CEI Hospital Civil de Guadalajara Fray Antonio Alcalde; Comite de Etica en Investigacion, Hospital no. 278 Col. Centro (Retiro), 44280, Guadalajara, Jalisco, Mexico.

OSMO, S.C.; Comité de ética en investigación y comité de investigación, Himboldt 302, col. centro, C.P. 68000, Oaxaca, Oaxaca, Mexico.

EC of The First Hospital of China Medical University, No.155, Nanjingbei Road, Heping District, Shenyang, 110001, Shenyang, China.

Yunnan Cancer Hospital; EC of Yunnan Cancer Hospital.

EC of Zhejiang Cancer Hospital; EC, No.38 Guangji Road, 310022, Hangzhou City, China.

EC of Jiangsu Province People Hospital, NO 300 Guangzhou Rd, 210029, Nanjing, China.

Comité de Ética en Investigación de la Facultad de Medicina y Hospital Universitario de la UANL, Av. Francisco I Madero y Av. Gonzalitos S/N, Col. Mitras Centro Monterrey Nuevo Leon, 64460, Monterrey Nuevo Leon, Nuevo Leon, Mexico.

Guangxi Medical University Cancer Center; EC of Guangxi Medical University Cancer Center, No. 71 Hedi Road, Naning, Guangxi, 530021, Nanning City, China.

Medical Ethics Committee of Union Hospital Tongji Medical College, No.1277 Jiefang Dadao, 430022, Wuhan City, China.

EC of Fujian Cancer Hospital, No. 420, Fuma Road, 350014, Fuzhou City, China.

Peking University People's Hospital; EC, No. 11, South Xizhimen Road, 100026, Beijing City, China.

EC of First Affiliated Hospital of Medical College of Xi'an Jiaotong University, No.277 Yanta West Road, 710061, Xi'an, China.

The Tumor Hospital of Xinjiang Medical University, No 789 east Suzhou street, 830000, Urumqi City, China.

Tianjin Cancer Hospital; EC of Tianjin Cancer Hospital, Floor 6, Building C Huanhu Road, Hexi District Tianjin China, China, 300060, Tianjin City, China.

Taipei Veterans General Hospital-Neurology; Institutional Review Board.

WIRB-Western Institutional Review Board, 1019 39th Avenue SE, Suite 120, Puyallup, WA, 98374, United States.

Egeszsegugyi Tudomanyos Tanacs - Klinikai Farmakologiai Etikai Bizottsaga, Alkotmany u.25, 1054, Budapest, Hungary.

Somogy Varmegyei Kaposi Mor Oktato Korhaz; Kutatasetikai Bizottsag, Tallian Gyula u. 20-34., 7400, Kaposvár, Hungary.

Ethical Clearance Committee on Human Rights, 270 RamaVI Road. Faculty of Medicine, Ramathibodi Hospital, Phayathai Rajathevi Bangkok 10400, 10400, Bangkok, Thailand.

ResearchEthics Com. Fac Med. Chiang Mai University, 110 Intavaroros Street, Amphoe Muang, 50200, Chaing Mai, Thailand.

Songklanagarind Ethics Committee, Prince of Songkla University, 15 Karnjanavanit Road, Hat Yai, 90110, Songkla, Thailand.

The Ethics Committee, Rajavithi Hospital, 2 Phyathai Rd. Rajthevee, 10400, Bangkok, Thailand.

US Oncology, Inc Institutional Review Board, 10101 Woodloch Forest, The Woodlands, TX, 77380, United States.

Comite de Etica en Investigacion Clinica (CEIC), Paraná 755, 6to. “A” y “B”, C1017AAO, Ciudad Autonoma Buenos Aires, Argentina.

Comité de Etica del Hospital Provincial del Centenario.

Comité de Ética en Investigación del Instituto de Oncología de Rosario.

Comité De Ética En Investigación en Salud – Fundación COIR, Montecaseros 1020, M5500AYB, Mendoza, Argentina.

Comitato Etico CESC dell’IRCCS Istituto Oncologico Veneto (IOV); Ospedale Busonera, Piazza Antenore, 3, 35121, Padova, Veneto, Italy.

Comitato Etico IRCCS Pascale, VIA M. SEMMOLA 1, 80131, NAPOLI, Campania, Italy.

Comitato Etico Policlinico Universitario A. Gemelli, Largo Agostino Gemelli 8, 00168, Roma, Lazio, Italy.

Comitato Etico della Provincia di Brescia, P.zza Spedali Civili, 1, 25123, Brescia, Lombardia, Italy.

CPP Sud Ouest Et Outre Mer I, 10 chemin du raisin, ARS Midi-Pyrénées, Bureau 1028, 31050, Toulouse cedex 9, France.

CEP ICAVC; Instituto do Câncer Dr. Arnaldo Vieira de Carvalho.

Comite De Etica Medica E Investigacion.

Budapesti Szent Margit Korhaz; Intezeti Tudomanyos es Kutatasetikai Bizottsag, BECSI UT 132., 1032, Budapest, Hungary.

The first affiliated hospital of Bengbu medical college; Ethics Committee of Bengbu Hospital, No.287, Changhuai Road, Bengbu, 233000, Bengbu City, China.

Comité de Etica Cayre.

# Appendices

## Biologic specimens

At participating sites, blood samples and human biologic samples for the Research Biosample Repository (RBR) will be collected from patients who give specific consent to participate in optional research. Blood samples will be used for whole genome sequencing or whole exome sequencing, to identify variants that are: predictive of response to study drugs; associated with progression to a more severe disease state; associated with acquired resistance to study drugs; associated with susceptibility to developing adverse events, can lead to improved adverse event monitoring or investigation, or can increase the knowledge and understanding of disease biology and drug safety.

RBR samples will be analyzed to achieve one or more of the following objectives: to study the association of biomarkers with efficacy or disease progression; to identify safety biomarkers that are associated with susceptibility to developing adverse events or can lead to improved adverse event monitoring or investigation; to increase knowledge and understanding of disease biology and drug safety; to study drug response, including drug effects and the processes of drug absorption and disposition; to develop biomarker or diagnostic assays and establish the performance characteristics of these assays.

Collection, storage, and analysis of RBR samples is contingent upon the review and approval of the exploratory research and the RBR portion of the Informed Consent Form by each site's IRB/EC and, if applicable, an appropriate regulatory body. RBR samples are to be stored until they are no longer needed or until they are exhausted. However, the RBR storage period will be in accordance with the institutional review board-/ethics committee-approved Informed Consent Form and applicable laws (e.g., health authority requirements).
